# Systematic Simulations of Structural Stability, Phonon Dispersions, and Thermal Expansion in Zinc-Blende ZnO

**DOI:** 10.3390/nano15040308

**Published:** 2025-02-17

**Authors:** Devki N. Talwar, Piotr Becla

**Affiliations:** 1 Department of Physics, University of North Florida, 1 UNF Drive, Jacksonville, FL 32224, USA; 2 Department of Physics, Indiana University of Pennsylvania, 975 Oakland Avenue, 56 Weyandt Hall, Indiana, PA 15705, USA; 3 Department of Materials Science and Engineering, Massachusetts Institute of Technology, Cambridge, MA 02139, USA; becla@mit.edu

**Keywords:** phonon dispersions, Debye temperature, specific heat, mode Grüneisen constant, thermal expansion, lattice dynamics, zinc-blende ZnO, thermal management

## Abstract

Zinc oxide (ZnO) has recently gained considerable attention due to its exceptional properties, including higher electron mobility, good thermal conductivity, high breakdown voltage, and a relatively large exciton-binding energy. These characteristics helped engineers to develop low dimensional heterostructures (LDHs)-based advanced flexible/transparent nanoelectronics, which were then integrated into thermal management systems. Coefficients of thermal expansion $\alpha \left(T\right),$ phonon dispersions $\;\omega_{j}(\vec{q})$, and Grüneisen parameters $\;\gamma_{j}\left(\vec{q}\right)$ can play important roles in evaluating the suitability of materials in such devices. By adopting a realistic rigid-ion model in the quasi-harmonic approximation, this work aims to report the results of a methodical study to comprehend the structural, lattice dynamical, and thermodynamic behavior of zinc-blende (zb) ZnO. Systematic calculations of $\omega_{j}(\vec{q})$, $\gamma_{j}\left(\vec{q}\right),$ and $\alpha \left(T\right)$ have indicated negative thermal expansion (NTE) at low T. Soft transverse acoustic shear mode gammas $\;\gamma_{TA}$ at critical points offered major contributions to NTE. Our results of $\omega_{j}(\vec{q})$ at ambient pressure compare reasonably well with Raman scattering spectroscopy measurements and first-principles calculations. By adjusting the layers of materials with positive and negative thermal expansion, it is possible to create LDHs with near-zero $\alpha \left(T\right)$. Such a nanostructure might experience a minimal dimensional change with T fluctuations, making it ideal for devices where precise dimensional stability is crucial.

## 1. Introduction

The wide bandgap (WBG) II-Oxides (II-Os: BeO, MgO, ZnO, and CdO) with $E_{g}$ (≡2.2–8.2 eV) have received considerable interest in recent years due to their spectacular physiochemical properties that are suitable for optoelectronic applications [1,2,3,4,5,6,7,8,9,10,11,12]. The ZnO material can be comparable to the wurtzite (wz) GaN, having similar characteristics to it, including a large direct bandgap $E_{g}$ $\left(≅3.37\;eV\right),$ and high breakdown voltage. Compared to the GaN and SiC materials, the ZnO has some advantages due to its higher ~ 60 meV exciton-binding energy and its availability as a large-sized single crystal [12]. Therefore, it has become the focus of global intensive research efforts to develop the most advanced flexible/ transparent nanoelectronics and photonics systems. These units are being integrated into high-power radio frequency modules, sensors/ biosensors, photodetectors/ solar cells, resistive random-access memory, and surface acoustic wave devices [13,14,15,16,17,18,19,20,21,22]. The high oscillator strength of ZnO is associated with a large amount of the exciton-binding energy, which might lead to the designing of lower-threshold lasers, with higher efficiency for faster optical switching and communication networks. Higher cohesive energy, as well as a high melting point with strong bonding, indicates that the degradation of ZnO-based devices during high temperature operations might not be an issue. More recently, the material has found another niche application for fabricating transparent thin-film transistors. These ZnO-based novel transistors [20,21,22,23,24,25,26] are insensitive to visible light where the protective covering preventing light exposure can be eliminated. 

Recent progress in bandgap engineering using II-Os is triggered by major improvements that have been made in controlling the growth process for achieving thin films with ionic polarities and intentional or unintentional doping. Syntheses of different materials (e.g., MgO-ZnO, BeO-MgO, ZnO-CdO) including their ternary and quaternary alloys have also contributed to the designing of various low dimensional heterostructures (LDHs) (e.g., multi-quantum wells (MQWs) and superlattices (SLs)). At heterointerfaces, the surface and/or interface properties of II-Os provided many interesting physical and chemical phenomena, such as the band bending, Fermi-level pinning, surface reconstruction, highly accumulated and/or depleted charge carriers, etc. The II-Os are generally categorized as highly mismatched materials due to the large difference in size and electronegativity between their metallic cation II and the O anion atoms [22,23,24,25,26]. These differences have resulted in many energetically favorable lattice defects (viz., cation/anion vacancies) for creating localized energy states in the energy bandgaps. Consequently, the ongoing research on novel ZnO material has offered many possibilities for discovering new physical phenomena and device concepts. 

The preparation of a large-scale cost-effective surface arrangement and functional II-O thin films has often demanded controlled design, patterns, and periodicity. These processes are essentially involved in the vaporization coating techniques, commonly referred to as the physical vapor deposition mechanisms, comprising the transfer of material at an atomic level onto a suitable substrate. Many epitaxial techniques have been adopted in recent years for growing the II-O films. The methods include the metal–organic chemical vapor deposition (MOCVD) [27,28,29], molecular beam epitaxy (MBE) [30,31,32,33,34,35], low-temperature solution-based microemulsion synthesis [36,37], solvothermal [38,39] oxford, and hydrothermal methods, etc. One must note that the growth of ZnO films does not require handling toxic ammonia, which is required in the fabrication of GaN-based device heterostructures [40,41,42,43,44,45,46,47,48,49,50,51,52]. 

Despite the successful efforts made by incorporating thin films and preparing different ZnO-based device structures, many fundamental issues have remained unresolved [22,23,24]. For instance, the structural, electronic, elastic, and vibrational properties have not been thoroughly investigated [53,54,55,56,57,58,59]. Another issue concerning ZnO has been the phase diagram, which is required for the metastable growth of zinc-blende (zb) structure in improving the optoelectronic devices. Similar condition played valuable role in preparing the zb GaN-based electronic structures and devices [41,42,43,44,45]. The zb ZnO has many superior features in terms of the lower carrier scattering, higher crystallographic symmetry, higher saturated electron drift velocity, and higher doping efficiencies, etc. For these reasons, the metastable zb polymorph is of great interest and is expected to solve certain problems associated with the wz and/or rock-salt (rs) structures [22].

The bulk ZnO material generally crystallizes in the wz or B_4_ form and undergoes a phase transition ($P_{t}$) to the rs structure or B_1_ type with pressure P ~ 8 GPa. Ultrathin ZnO films prepared on GaAs substrate have exhibited the zb cubic or B_3_ phase. Within the first-principle molecular dynamics (MD) simulations, Wu and Kang [60] have predicted a possible phase transition from B_4_ → B_3_ at $P_{t}$ ~ 26 GPa. Their estimated value of $P_{t}$ differred considerably from other theoretical results [22,59]. It is to be noted that the stable crystallographic phases are highly associated with many extrinsic factors, including the compositional fluctuations and heterogeneities [22]. For the preference of stability requirements, appropriate conditions, such as high T, high-pressure P, and heavy dopant concentrations, are necessary for achieving the metastable structures [22,56,57]. The zb ZnO/GaAs epifilms have already been prepared [56,57]. Additionally, the successful growth of binary thin films and using alloy epilayers for creating a variety of configurations in the layered MQWs, and SLs have resulted in endless flexibility of designing and fabricating electro-optic devices. Thus, it is important to explore experimentally and theoretically by carefully characterizing/evaluating the elastic, structural, and phonon properties of II-Os for assessing their basic features using the physics/chemistry of chemical bonding. 

Although wz binary materials have been used in many practical/industrial and technological applications, less attention is paid to the zb polymorph despite the successful growth of ultrathin epifilms [56,57]. A variety of characterization techniques have also been applied to analyze/monitor their fundamental properties [61,62,63,64,65,66,67,68,69,70,71,72,73,74,75,76,77,78,79,80,81,82,83]. Different methods are used in the classification, which include the reflection high-energy electron diffraction (RHEED) [61,62], Auger electron spectroscopy (AES) [63], He^+^ Rutherford backscattering spectrometry (RBS) [64], atomic force microscopy (AFM) [65,66], high-resolution X-ray diffraction [66,67] (HR-XRD), transmission electron microscopy (XTEM) [67,68,69,70,71], photoluminescence (PL) [72], absorption, Fourier transform infrared (FTIR) spectroscopy [74], Raman scattering spectroscopy (RSS) [75,76,77,78,79,80,81], spectroscopic ellipsometry (SE) techniques [82,83], etc. It is to be noted that not only have these methods validated the crystal structures, but they have also helped in evaluating the epifilm thickness, strain, intrinsic electrical, and optical traits. Despite an extensive use of different studies for assessing the structural and electrical properties of wz materials, there are limited IR absorption and RSS measurements [80] available on the zb ZnO/GaAs films for appraising their phonon and bonding characteristics. 

From a theoretical standpoint, several calculations are performed to understand the structural, electronic, and optical features of the binary wz ZnO material by using a full-potential linear-augmented plane wave (FP-LAPW), first-principles (ab initio), and MD methods [84,85,86,87,88,89,90,91,92,93,94,95,96,97,98]. Very few calculations are known, however, for the zb ZnO phase. Moreover, some of the reported results on the electronic and vibrational characteristics are either inconsistent and/or questionable [91,92]. For instance, Chibueze [91] adopted an ab initio approach to study the mechanical, phonon, and electronic properties of zb ZnO in the framework of density function theory (DFT), using a generalized gradient approximation (GGA) by considering a revised PBEsol (Perdew–Burke–Ernzerhof GGA) method. The author [91] claimed to have achieved the degenerate phonon energy values at the center of the Brillouin zone (BZ) (i.e., near ${\vec{q}\to 0;\omega }_{LO\left(\Gamma \right)}=\omega_{TO\left(\Gamma \right)}\;\sim \;379\;{cm}^{-1}).$ This result is in complete disagreement with the phonon dispersions $\omega_{j}(\vec{q})$ reported experimentally and theoretically by Serrano et al. [80] for the zb ZnO material and the LDA simulation [89]. The pressure-induced changes in the elastic, electronic, and vibrational properties of wz ZnO have also been accomplished using the commercially available SIESTA and ABIN software packages [84,85,86,87,88,89,90,91,92,93,94,95,96,97,98]. For the ternary X_x_Y_1−x_O alloys, no systematic studies are known for comprehending their lattice dynamical and/or thermodynamic characteristics. There are many expected heteroepitaxial growth problems caused by lattice mismatch, which include the difference in thermal expansion coefficients, cross doping, interface polarity, and phase segregations. These issues have been and still are considered to be the key obstacles for achieving high quality heteroepitaxial device structures. Although the first-principles study of the negative thermal expansion coefficient of wz and zb ZnO material has been reported by Wang et al. [95], using DFT in the quasi-harmonic approximation (QHA), for zb ZnO, no Grüneisen parameter values are displayed for the optical phonons. Moreover, the calculations of Debye temperature $\Theta_{D}(T)$ and specific heat $C_{v}\left(T\right)$, which are closely linked to the thermal expansion α(T), are also missing [95]. To accomplish the major technological applications of different heterostructures, systematic calculations are necessary to obtain the complete phonon dispersions $\omega_{j}(\vec{q})$ of the binary ZnO (BeO, MgO, CdO) and ternary alloys, Zn_x_Cd_1−x_O, Zn_x_Mg_1−x_O, and Be_x_Cd_1−x_O, from realistic lattice dynamical models. One reason for this prerequisite is that the dynamical response of polar lattices affects the key electronic properties, including the exciton-binding energy and the charge carrier mobilities. Examining the dynamical response of crystals and their impact on dielectric traits provides a major step forward in realizing their structural characteristics. The other reason for its need is that, in MQWs and SLs, the phonon density of states (DOS) of the binary/ternary alloys has always played a crucial role in evaluating the thermodynamic traits, including α(T), $\Theta_{D}$(T), $C_{v}(T)$, Grüneisen constants γ(T), entropy, and lattice thermal conductivity, etc. 

This paper aims to present results of a methodical study based on a realistic rigid-ion model (RIM) [99] for comprehending the structural, lattice dynamical, and thermodynamic characteristics of the zb ZnO material. The procedure that we followed in this work has been succinctly outlined in Section 2.2, Section 2.2.1, Section 2.2.2, Section 2.2.3 and Section 2.2.4 with the detailed descriptions reported elsewhere [100]. Murnaghan’s equation of state [101] is adopted (cf. Section 3, Section 3.1 and Section 3.1.1) for assessing the P-dependent volume ($V/V_{0}$) or lattice constant ratio ($a/a_{0}$) of the zb ZnO, using the appropriate bulk modulus $B_{0}$ and pressure derivative $B_{0}^{\prime }.$ Novel optimization procedures [100] are implemented for estimating the RIM interatomic force constants (IFCs) at ambient pressure, i.e., at 1 atm or P = 0 GPa and P = 8 GPa, by carefully incorporating experimental critical point phonon energies as input, while using lattice constant $a_{0}$, elastic constants $c_{ij}$, and their pressure derivatives $\partial c_{ij}/\partial P$ [100] as constraints. In Section 3.1 and Section 3.2, we have reported the results of phonon dispersion relations $\omega_{j}(\vec{q})$ at ambient and high pressure, as well as a detailed analysis of one-phonon g(ω) and two-phonon $g_{2}^{\pm }(\omega )$ DOS. These results are required for successful interpretations of the P-dependent second-order experimental observations in the RSS studies [77,78,79,80]. The simulated values (cf. Section 3.3 and Section 3.3.1) of pressure coefficients $\partial \omega_{j}(\vec{q})/\partial P$ are integrated for achieving the mode Grüneisen dispersions $\gamma_{j}(\vec{q})$ at critical points in the Brillouin zone (BZ). Some selected thermal properties obtained in the QHA [e.g., Debye temperature $\;\Theta_{D}\left(T\right),$ heat capacity at constant volume $C_{v}(T)$, Grüneisen parameter γ(T), and thermal expansion coefficient α(T)] are compared to the available experimental/theoretical data. For zb ZnO, the quantitative analysis of γ(T) and α(T) has confirmed our earlier assertion [102] that the zone-edge $\omega_{TA}$ modes become negative due to P-induced softening of the bond-angle-bending force constants for causing negative thermal expansion (NTE) at low T. We have compared/contrasted and discussed our systematically investigated structural, phonon, and thermodynamical properties of zb ZnO against the existing experimental [80,103,104,105] and first-principles calculations [89,95,98], with concluding remarks presented in Section 4.

## 2. Theoretical Background

### 2.1. Crystal Structure

**Figure 1:**
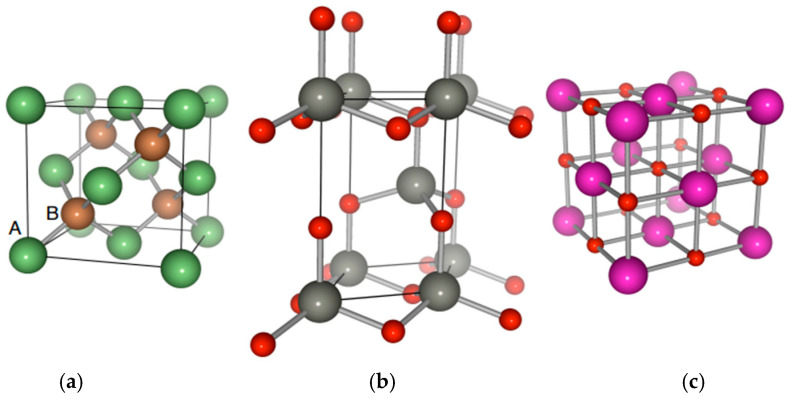
Schematic representation of the crystalline structures of ZnO. (**a**) Cubic zinc-blende B_3_ phase, where the large green colored (A) and small brown colored (B) spheres represent, respectively, the atoms of zinc and oxygen, (**b**) hexagonal wurtzite B_4_ structure where the large grey and small red color spheres represent, respectively the zinc and oxygen atoms, and (**c**) cubic NaCl-like rs B_1_ structure, where the large violet and small red color spheres represent, respectively the zinc and oxygen atoms.

The ultrathin ZnO films with a space group of $F\bar{4}3m\left(T_{d}^{2}\right)$ can exhibit the zb cubic phase or B_3_ structure if prepared on GaAs and/or Si substrates [56,57] (see Figure 1a) that have a mixture of tetrahedral covalent and ionic bonding. For the ambient conditions of temperature T and pressure P, the ZnO semiconductor belongs to a $P6_{3}mc(C_{6v}^{4})$ space group (see Figure 1b), and usually occurs in the hexagonal wz or B_4_ structure. At high pressure, the material can crystallize in the NaCl-like rs or B_1_ polymorph (see Figure 1c) with a space group, $Fm\bar{3}m(O_{h}^{5})$. For most substrates, the epitaxially grown ZnO yields a stable wz structure; however, there are reports of achieving epifilms with the zb crystal structure when prepared on Si or GaAs substrates [56,57]. 

#### Phase Transitions

Experimental studies of P-induced structural phase transitions in semiconductors started in the early 1960s. These investigations have gained considerable interest in recent years due to the improved diamond-anvil cell (DAC) and progress made in using different optical and X-ray measurements, with significant advances achieved in controlling the appropriate P-range [22]. In ZnO materials, the phase transition mechanism under high P showed many unique features and is still under exploration. In most semiconductors, the values of transition pressure $P_{t}\;$ (see Table 1) are frequently assessed by using either the DAC, HR-XRD, RSS, and/or imaging-plate techniques. However, it has been, and still is, quite a challenge for experimentalists to find an accurate equilibrium $P_{t}$, i.e., P, where both of the phases of materials coexist in equilibrium. Hysteresis due to an energy barrier is common in the first-order phase transition, making it hard to locate the transition point [22]. Furthermore, the value of $P_{t}$ could be sensitive to measurement methods, sample type, and non-hydrostatic stress conditions.

Studying the structural phase transition in solids also poses considerable challenges for the theorists using different models. The high-pressure status in ZnO has been and still is extremely ambiguous [84,85,86,87,88,89,90,91,92,93,94,95,96,97,98]. Consequently, many existing experimental and theoretical studies have offered large discrepancies in their $P_{t}$ values. Several DFT simulations in ZnO have provided structural changes from B_4_ → B_3_ → B_1_ phases, predicting different values (2 GPa < $P_{t}$ < 26 GPa), with an average $P_{t}$ of ~8 GPa for the ZnO. In Table 1, we have listed the calculated equilibrium $P_{t}$ in GPa for ZnO by different researchers using various ab initio methods [84,85,86,87,88,89,90,91,92,93,94,95,96,97,98].

From the application standpoint, II-Os exhibit interesting similarities to III-Ns. For developing blue LEDs and lasers, the ZnO is considered an alternative to GaN. Achieving stable p-type doping remained, however, as the most daunting obstacle in producing bipolar ZnO-based devices [12]. For exploiting excitonic transitions to design lasers, ZnO would certainly be superior to GaN, provided that p-type conductivity is achieved, and other necessary processing capabilities are developed for the ZnO [1,2,3,4,5,6,7,8,9,10,11,12]. Due to different mechanical and thermodynamical properties [106], considering ZnO/BeO-based LDHs would certainly be a valuable step forward for thermal management applications [1,2,3,4,5,6,7,8,9,10,11,12]. 

### 2.2. Computational Methodology

Two theoretical approaches are commonly used to comprehend the lattice dynamics of zb materials, which are as follows: (i) the microscopic or first-principles methods [84,85,86,87,88,89,90,91,92,93,94,95,96,97,98], which start with an ionic potential screened by the electron gas for deriving the structural and vibrational properties, and (ii) the macroscopic technique, which employs the phenomenological models [99,100] in terms of the general interatomic forces. For the zb ZnO, we have systematically obtained the results of phonon dispersions at P = 1 atm (or 0 GPa) and P = 8 GPa by exploiting a realistic RIM [99] (cf. Section 2.2.1).

#### 2.2.1. Rigid-Ion Model

Phonon dispersion calculations for the zb ZnO are performed in the framework of a rigid-ion model developed by Kunc [99]. In the harmonic approximation, the crystal Hamiltonian can be expressed as follows:

(1) $$H=\sum_{l\kappa \alpha }\frac{p_{\alpha }^{2}(l\kappa )}{2M_{\kappa }}+\Phi_{0}+\frac{1}{2}\sum_{l\kappa \alpha ,l\prime \kappa \prime \beta }\Phi_{\alpha \beta }(l\kappa ,l\prime \kappa \prime )u_{\alpha }(l\kappa )u_{\beta }(l\prime \kappa \prime ),$$

where $u_{\alpha }(l\kappa )$ is the α-component of the displacement of the κth (≡1, 2)-type atom from equilibrium in the lth unit cell, and $p_{\alpha }\left(l\kappa \right)$ is the corresponding component of the momentum. For zb crystals, one can write the potential energy $\Phi_{\alpha \beta }(l\kappa ,l\prime \kappa \prime )$ by splitting it into a short-range repulsive and a long-range Coulomb part, as follows [99]:

(2) $$\Phi_{\alpha \beta }\left(l\kappa ,l\prime \kappa \prime \right)=\Phi_{\alpha \beta }^{s}\left(l\kappa ,l\prime \kappa \prime \right)+Z_{\kappa }Z_{\kappa \prime }\Phi_{\alpha \beta }^{C}(l\kappa ,l\prime \kappa \prime ),$$

with $Z_{\kappa }$e being the charge on the $\kappa th\left(\equiv 1,2\right)$-type ions.

The equations of motion take the following form [99]:

(3) $$M_{\kappa }{\ddot{u}}_{\alpha }\left(l\kappa \right)=-\sum_{l\prime \kappa \prime \beta }\Phi_{\alpha \beta }(l\kappa ,l\prime \kappa \prime )u_{\beta }(l\prime \kappa \prime ).$$

One can express the atomic displacement as a plane wave of the type

(4) $$u_{\alpha }\left(l\kappa |\vec{q}j\right)=\frac{1}{\sqrt{M_{\kappa }}}e_{\alpha }\left(\kappa |\vec{q}j\right)e^{i\left[\vec{q}\vec{x}\left(l\kappa \right)-\omega_{j}\left(\vec{q}\right)t\right]};\;with\;\alpha =x,y,z,$$

where t identifies the time; $\vec{x}\left(l\kappa \right)$ and $M_{\kappa }$ represent, respectively, the position and mass of the (lκ) atom. By substituting Equation (4) into Equation (3), it is possible to write the equations of motion as follows [99]:

(5) $$\omega_{j}^{2}(\vec{q})e_{\alpha }(\kappa |\vec{q}j)=\sum_{\kappa^{\prime }\beta }D_{\alpha \beta }^{sC}(\kappa \kappa \prime |\vec{q})e_{\beta }(\kappa \prime |\vec{q}j);\kappa ,\kappa \prime =1,2$$

where $D_{\alpha \beta }^{sC}(\kappa \kappa \prime |\vec{q})\left[\equiv D_{\alpha \beta }^{s}(\kappa \kappa \prime |\vec{q})+D_{\alpha \beta }^{C}(\kappa \kappa \prime |\vec{q})\right]$ represents the dynamical matrix comprising the short- $D_{\alpha \beta }^{s}(\kappa \kappa \prime |\vec{q})$ and long-range Coulomb $D_{\alpha \beta }^{s}(\kappa \kappa \prime |\vec{q})$ interactions. For each mode $\omega_{j}(\vec{q})$ the components of eigenvectors $e_{\alpha }(\kappa |\vec{q}j)$ in Equation (5) satisfy the familiar orthogonality [99,100]:

(6a) $$\sum_{\alpha \kappa }e_{\alpha }^{*}(\kappa |\vec{q}j)e_{\alpha }(\kappa |\vec{q}j\prime )=\delta_{jj\prime },$$

and closure relationships

(6b) $$\sum_{j}e_{\alpha }^{*}(\kappa \prime |\vec{q}j)e_{\beta }(\kappa |\vec{q}j\prime )=\delta_{\kappa \kappa \prime }\delta_{\alpha \beta }.$$

Once the interatomic RIM force constants $A,B,C_{\kappa },D_{\kappa },E_{\kappa },F_{\kappa },$ and effective charge $Z_{eff}(\equiv Z_{\kappa }e)$ for the zb ZnO material (cf. Section 3.1.2) are evaluated, it is straightforward to simulate $\omega_{j}(\vec{q})$ by using Equation (5).

#### 2.2.2. The Quasi-Harmonic Approximation

The temperature dependence of lattice parameter $a_{0}$ can be measured experimentally by HR-XRD. Along with thermal conductivity κ(T), the specific heat $C_{v}(T)$, thermal expansion coefficient $\alpha \left(T\right),$ and Grüneisen parameters γ(T) are the other three most important characteristics that can contribute to the fundamental understanding of the lattice anharmonicity and help to assess the utility of a material in thermal management applications [84,85,86,87,88,89,90,91,92,93,94,95,96,97,98]. To measure the specific heat of a semiconductor, researchers have typically used a technique called differential scanning calorimetry. In this method, a small sample of a semiconductor is heated at a controlled rate, which compares the heat flow to a reference material, allowing for the calculation of a specific heat capacity based on temperature change and the heat absorbed by the sample. The thermal expansion coefficient can be measured in a three-terminal capacitance dilatometer [103,104,105]. Furthermore, except for the wz ZnO, no such experiments of $\alpha \left(T\right)\;$ are known for the zb ZnO material.

Theoretically, the knowledge of IFCs between the atoms of zb ZnO is required to obtain and solve the equations of motion in the harmonic approximation (cf. Section 2.2.1). The inclusion of higher terms in Equation (1) makes the exact solutions to the equations of motion impossible, and approximations must be employed. For a system that is only slightly anharmonic, the QHA is frequently used. In this approximation, the frequencies of phonon modes are considered to depend only on T and P via the volume change, i.e., through a change in lattice spacing $a_{0}$. The interatomic potential is still terminated in the quadratic term, allowing for the definition of normal modes, as with the harmonic approximation; however, now the IFCs are allowed to change with the change in interatomic distance. Hence, the phonon dispersions $\omega_{j}(\vec{q})$ and density of states DOS will shift with the change in volume (or pressure), be it due to thermal expansion or by an externally applied stress (pressure).

#### 2.2.3. Thermal Properties 

The results of RIM lattice dynamics methodology have been extended to evaluate different thermodynamic quantities, including $\Theta_{D}\left(T\right),$ $C_{v}(T)$, γ(T), and $\alpha \left(T\right)$, in the QHA. It is to be noted that the anharmonic effects are included in the QHA by considering the volume (pressure) dependence of the phonon modes. Again, the phonon frequencies at constant volume are assumed to be independent of T. Moreover, the relative change in the phonon ($\vec{q}j$) mode frequency $\omega_{j}(\vec{q})$ with volume V (or P) is usually described by the mode-specific Grüneisen dispersion $\gamma_{j}\left(\vec{q}\right),$ which is a dimensionless quantity defined as follows [102]:

(7) $$\gamma_{j}\left(\vec{q}\right)=-\frac{d\left(ln\omega_{j}\left(\vec{q}\right)\right)}{dlnV}=-\frac{V}{\omega_{j}\left(\vec{q}\right)}\frac{d\omega_{j}\left(\vec{q}\right)}{dV}=\frac{B_{0}}{\omega_{j}\left(\vec{q}\right)}\frac{d\omega_{j}\left(\vec{q}\right)}{dP},$$

where $B_{0}$ is the bulk modulus. The above Equation (7) serves to quantify the vibrational anharmonicity. The positive sign of $\gamma_{j}\left(\vec{q}\right)\;$ infers that the phonon frequencies are increasing with the decrease in volume (increase in pressure), suggesting a positive thermal expansion coefficient. A negative $\gamma_{j}\left(\vec{q}\right)\;$ is an indication of the soft mode behavior. The relevance of negative Grüneisen parameters and soft modes to the NTE in zb ZnO will be discussed in Section 3.3, Section 3.3.1, Section 3.3.2, Section 3.3.3 and Section 3.3.4. For an isotropic crystalline solid, the thermal expansion coefficient α in the QHA can be expressed as:

(8) $$\alpha =\frac{1}{3B_{0}}\sum_{\vec{q},j}C_{v}\left(\vec{q},j\right)\gamma_{j}\left(\vec{q}\right),$$

Using $\gamma_{j}\left(\vec{q}\right),$ the thermal Grüneisen parameter γ(T) can be obtained by using the relationship [102]:

(9) $$\gamma \left(T\right)=\frac{\sum_{\vec{q},j}\gamma_{j}\left(\vec{q}\right)C_{v}\left(\vec{q},j\right)}{\sum_{\vec{q},j}C_{v}\left(\vec{q},j\right)},$$

where the contribution of each mode $\omega_{j}(\vec{q})$ to $\gamma \left(T\right)\;$ is weighted by its contribution to the specific heat $C_{v}\left(\vec{q},j\right)$. 

The denominator in the above Equation (9) is the heat capacity at constant volume, which can take the following form [100]:

(10) $$C_{v}(T)=k_{B}\sum_{\vec{q},j}{\left(\frac{\hbar \omega_{j}\left(\vec{q}\right)}{2k_{B}T}\right)}^{2}\frac{1}{{sinh}^{2}\left(\frac{\hbar \omega_{j}(\vec{q})}{2k_{B}T}\right)},$$

where T is the temperature; $k_{B}$ and ħ are, respectively, the Boltzmann and Planck constants. It is also possible to express $C_{v}$ via the calculated phonon density of states g(ω). Thus, an equivalent form of Equation (10) can be rewritten as follows [100]:

(11) $$C_{v}(T)={Nrk}_{B}\int_{0}^{\infty }d\omega g(\omega )\left(\frac{\hbar }{k_{B}T}\right)\frac{exp\left(\frac{\hbar \omega_{j}(\vec{q})}{k_{B}T}\right)}{{\left[exp\left(\frac{\hbar \omega_{j}(\vec{q})}{k_{B}T}\right)-1\right]}^{2}}$$

By using Equations (9)–(11), the T-dependence of the Grüneisen constant can be evaluated, in the following manner [102]:

(12) $$\gamma \left(T\right)=\frac{\sum_{\vec{q},j}\gamma_{j}\left(\vec{q}\right)C_{v}\left(\vec{q},j\right)}{C_{v}(T)}.$$

Again, from the Debye’s equation [100], $C_{v}(T)$ can also be expressed as follows:

(13) $$C_{v}(T)={9rk}_{B}{\left(\frac{T}{\Theta_{D}(T)}\right)}^{3}\int_{0}^{\Theta_{D}(T)/T}\frac{x^{4}e^{x}}{{\left(e^{x}-1\right)}^{2}}dx,$$

Once the complete phonon dispersions $\omega_{j}\left(\vec{q}\right)\;and$ Grüneisen dispersions $\gamma_{j}\left(\vec{q}\right)$ are obtained for the wave vectors throughout the BZ, the T-dependent Debye temperature of $\Theta_{D}\left(T\right),$ $\gamma \left(T\right)$, or $\alpha \left(T\right)$ can be easily simulated.

#### 2.2.4. Effective Charge and Its Pressure Dependence

In polar materials, the Born’s transverse effective charge $e_{T}^{*}$ is defined as a variation of the force on a given atom under an applied electric field. It is the key parameter used for understanding the coupling between the lattice vibrations and the electric field. In a majority of simple diatomic crystals, including the zb or wz III-V, II-VI, and I-VII semiconductors, the frequencies of long wavelength transverse optic TO ($\omega_{TO(\Gamma )}$) and longitudinal optical LO ($\omega_{LO(\Gamma )}$) modes are known, along with the low- and high-frequency dielectric constants ($\varepsilon_{0}$, $\varepsilon_{\infty }$). 

In such materials, the oscillator strength of the TO phonon is reflected in the difference between squares of their $\omega_{LO(\Gamma )}$ and $\omega_{TO(\Gamma )}$ modes, or, alternatively, via the so-called Lyddane–Sachs–Teller (LST) relationship $\left(\frac{\omega_{LO}^{2}}{\omega_{TO}^{2}}=\frac{\varepsilon_{0}}{\varepsilon_{\infty }}\right).$ It is commonly described by one of the two parameters, the Born’s $e_{T}^{*}$ or the $Szigeti’se_{S}^{*}[=3e_{T}^{*}/\varepsilon_{\infty }+2]$ effective charge. Among these two effective charges, $e_{T}^{*}$ is model-independent and can be calculated from the readily observable quantities by using the following equation [79]:

(14) $$\omega_{LO(\Gamma )}^{2}-\omega_{TO\left(\Gamma \right)}^{2}=\frac{4\pi {(e}_{T}^{*}{)}^{2}N}{\varepsilon_{\infty }\mu Ω},$$

where the high-frequency or optical (electronic) dielectric constant $\varepsilon_{\infty }$ is for phonon frequencies well-above the $\omega_{LO}$ but below the optical absorption edge, i.e., the dielectric constant in the absence of lattice vibrations, and μ is a reduced mass. With an explicit use of the LST relation, the above Equation (14) can take the following form [79]:

(15) $$\varepsilon_{0}=\varepsilon_{\infty }+\frac{4\pi {(e}_{T}^{*}{)}^{2}N}{\mu \omega_{TO\left(\Gamma \right)}^{2}}=\varepsilon_{\infty }+\varepsilon_{lat}.$$

Here, $\varepsilon_{lat}$ is the lattice contribution to the dielectric constant. In heteropolar semiconductors, the term $\varepsilon_{lat}$ arises from the fact that the $\omega_{LO(\Gamma )}$ mode produces a macroscopic electric moment which separates it in frequency from the $\omega_{TO(\Gamma )}$ phonon. The homopolar Si crystal with the center of inversion symmetry between atoms causes $\omega_{LO\left(\Gamma \right)}=\omega_{TO\left(\Gamma \right)}$. Thus, the lattice vibrations make no contribution to $\varepsilon_{0};$ $\varepsilon_{0}=\varepsilon_{\infty }=n^{2}$, with n being the index of refraction.

From Equation (15), it is quite evident that $\varepsilon_{lat}$ and its P-dependence can be derived by using the following relation [79]:

(16) $$\frac{{\partial ln\varepsilon }_{0}}{\partial P}=\frac{\varepsilon_{\infty }}{\varepsilon_{0}}\frac{{\partial ln\varepsilon }_{\infty }}{\partial P}+\frac{\varepsilon_{lat}}{\varepsilon_{0}}\frac{{\partial ln\varepsilon }_{lat}}{\partial P},$$

provided that the values of $\varepsilon_{0}$, $\varepsilon_{\infty }$, and their respective pressure variations are known. Once the pressure dependence of $\varepsilon_{lat}$ is obtained, one can easily predict the effect of compression on the covalency (ionicity or Born’s effective charge $e_{T}^{*}$) of the polar semiconductor bond, as follows [cf. Equation (15)] [79]:

(17a) $$\frac{\partial lne_{T}^{*}}{\partial P}=\frac{1}{2}\frac{\partial ln\varepsilon_{lat}}{\partial P}+\frac{\partial ln\omega_{TO\left(\Gamma \right)}}{\partial P}-\frac{1}{2}\chi_{0},$$

or

(17b) $$\gamma_{e_{T}^{*}}=\frac{1}{2}\gamma_{lat}+\gamma_{TO\left(\Gamma \right)}-\frac{1}{2}\;,$$

where $\chi_{0}\left(\equiv \frac{1}{B_{0}}\right)$ is the volume compressibility and $\gamma_{e_{T}^{*}}$ is known as the Grüneisen parameter for the Born’s dynamic charge. The effect of pressure on the bonding mechanism in semiconductors may also be learned from the reliable lattice dynamical calculations (cf. Section 3).

## 3. Numerical Computations, Results, and Discussion

### 3.1. Determination of Parameters

To see how the Grüneisen constants, $\gamma \left(T\right)$ and $C_{v}\left(T\right),$ can be used to simulate $\alpha \left(T\right)$ in the QHA, it is necessary to assess (cf. Section 3.1.2) the unique sets of eleven RIM interatomic force constants, $a_{i}\left(i=1\;to\;11\right),$ both at the ambient (P = 0 GPa) and high pressure (P ≠ 0 GPa). 

#### 3.1.1. Structural and Elastic Properties of zb ZnO

To calculate the P-dependent lattice constants of the zb ZnO material, we have adopted an extrapolated Murnaghan’s equation of state, which can be expressed in the following way [101]:

(18) $$\frac{a}{a_{0}}={\left[\frac{B_{0}^{\prime }}{B_{0}}P+1\right]}^{-1/3B_{0}^{\prime }},$$

where $B_{0}^{\prime }$ is the pressure derivative of the bulk modulus $B_{0}$.

**Figure 2:**
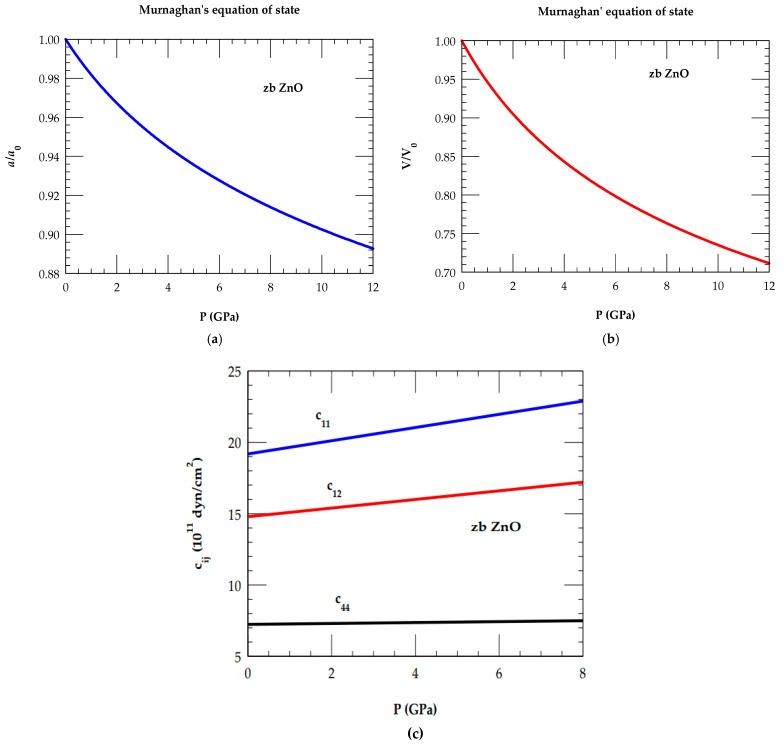
(**a**) Calculated variation of lattice constant ratio $\frac{a}{a_{0}}\;$; (**b**) volume ratio $\frac{V}{V_{0}}$ as a function of pressure P for zb ZnO based on Murnaghan’s equation of state Ref. [101] by using parameter values from Table 2; and (**c**) calculated variations of the elastic constants of $c_{11}$, $c_{12}$, and $c_{44}$ as a function of P, satisfying the mechanical stability conditions, viz., ($c_{11}-c_{12}$) > 0, ($c_{11}+2c_{12}$) > 0, and $c_{44}>0$.

For zb ZnO, the appropriate physical parameters listed in Table 2 are employed in Equation (18) for calculating the lattice constant ratio $\frac{a}{a_{0}}$. In Figure 2a,b, the simulated results of P-dependent variations for $\frac{a}{a_{0}}$ (volume ratio $\frac{V}{V_{0}})$ are displayed between the P (≡ 0 → 12 GPa). Figure 2c shows that in zb ZnO, the elastic constants $c_{ij}$ satisfy the mechanical stability conditions.

#### 3.1.2. Rigid-Ion Model Parameters of zb ZnO

At ambient P, the IFCs of RIM (e.g., $A,B,C_{\kappa },D_{\kappa },E_{\kappa },F_{\kappa }$, and $Z_{eff})$ are systematically optimized (see Table 3) by using the elastic constants $c_{ij}$, critical point phonon frequencies, and lattice parameters $a_{0}$ (see Table 2). To obtain the P-dependent IFCs (see Table 3), we used phonon energies derived either from the high-pressure Raman scattering spectroscopy measurements [80] and/or reliable theoretical schemes as the input and considered the simulated pressure-dependent elastic $\frac{\partial c_{ij}}{\partial P}$ (see Figure 2c), and lattice $\frac{\partial a}{\partial P}$ constants (see Figure 2a) as constraints. 

Like other III-V and II-VI compound semiconductors, the RIM interatomic force constants of zb ZnO material are evaluated systematically by using successive least-squares fitting procedures described in detail elsewhere [100]. In these methods, we incorporate either the experimental (cf. Table 2) and/or reliable first-principles data of phonons at high critical points in the BZ as the input, while employing the elastic $c_{ij}$ and the equilibrium lattice constant a_o_ as constraints. 

In Table 3, we have listed our calculated values of IFCs for the zb ZnO material at ambient pressure P = 0 and high P = 8 GPa. The perusal of Table 3 has clearly revealed significant changes in the values of IFCs. To realize the importance of the two sets of model parameters and for evaluating the structural, lattice dynamical, and thermodynamic properties of zb ZnO material at any pressure P, we have considered a linear interpolation scheme, which is as follows [102]:

(19) $$a_{i}\left(P\neq 0\right)=a_{i}\left(P=0\right)+P\frac{da_{i}}{dP},$$

where $a_{i}$ (i = 1, 11) represents the values of an optimized set of 11 RIM parameters, reported in Table 3. 

### 3.2. Lattice Dynamics

Using the IFCs from Table 3 and Equation (19), one can calculate the pressure-dependent phonon properties for zb ZnO at any desired P. 

#### 3.2.1. Phonon Dispersions and One-Phonon Density of States

**Figure 3:**
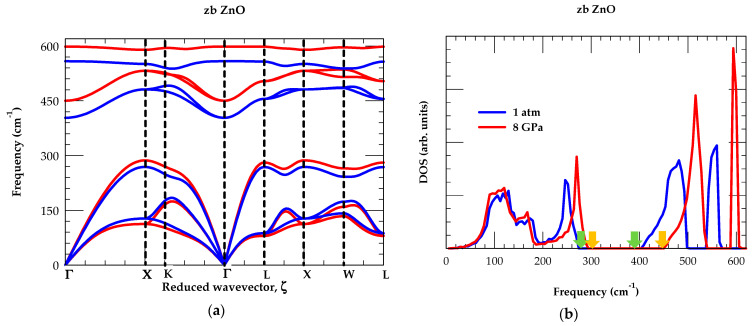
(**a**) Rigid-ion model calculations of the phonon dispersions for zinc-blende ZnO material where the blue- and red-color lines represent our results at ambient P = 0, and P = 8 GPa, respectively; (**b**) Rigid-ion model calculations of the one-phonon density of states. The vertical green- and orange-colored arrows represent the edges of the phonon gaps at P = 0 and P = 8 GPa, respectively.

For zb ZnO material, the results of our RIM calculations for $\omega_{j}(\vec{q})$ at ambient P (blue color) and P = 8 GPa (red color) lines are displayed in Figure 3a, along the high symmetry directions (Γ → X → K → Γ → L → X → W → L) of the BZ. The simulated one-phonon DOS $g\left(\omega \right),$ at P = 0 and P = 8 GPa, are also reported in Figure 3b. 

In Table 4, we have reported and compared our RIM results of phonon frequencies at high critical points (Γ, X, L, K, W) (cf. Figure 3a) against the existing experimental [80] and/or first-principles calculations [89]. The inspection of Figure 3a,b has confirmed that the optical and acoustical phonon modes of zb ZnO material are affected by the atomic masses of light O (16.0 amu) and heavier Zn (65.38 amu) atoms, respectively. It is to be noted that the first and second peaks in the low-frequency region of the DOS (see Figure 3b) are associated with the average $\omega_{TA}$ and $\omega_{LA}$ modes, while the two peaks in the high-frequency optical phonon energy region are linked to the average $\omega_{TO}$ and $\omega_{LO}$ phonons, respectively. At ambient pressure (i.e., P = 0 GPa), our study has revealed that a phonon gap in zb ZnO occurs between the maximum acoustic modes and the minimum optical phonons region at ~275–405 cm^−1^ (shown by the light green-color vertical arrows), which shifts to the higher frequency region between ~290–454 cm^−1^ for P = 8 GPa (indicated by the orange-color vertical arrows). Again, from $g\left(\omega \right)$ at P = 8 GPa (red-color lines), the $\omega_{TA}$ modes shift towards the lower frequency section (cf. Figure 3a), causing negative $\frac{\partial \omega_{TA}}{\partial P}\;$(cf. Table 4). All other phonons have exhibited positive values, i.e., $\frac{\partial \omega_{j}\left(\vec{q}\right)}{\partial P}$.

For zb ZnO, the calculated trends in the P-dependent results (Figure 3a,b) of phonon dispersions, and at high critical points in the BZ, can be summarized more precisely as follows: (i) the frequencies of the lowest TA branches at or near the zone-boundary decrease or soften with the increase in pressure, P, including the predicted shifts of $\omega_{TA(X)}$ and $\omega_{TA(L)}$ phonon modes at X- and L-critical points, with large $\vec{q}$ values that are typical of this effect; (ii) the energies of LA-phonons (i.e., $\omega_{LA(X)}$ and $\omega_{LA(L)}$) increase with P; and (iii) the optical phonons at the zone center (Γ point} of the BZ shift to higher energies with an increase in P. These observations agreed reasonably well with our earlier outcomes on the II-VI and III-V compound semiconductors [102].

One must also note that our RIM study has predicted the correct optical phonon splitting $\Delta \omega_{opt}$(≡$\omega_{LO(\Gamma )}$ − $\omega_{TO(\Gamma )}$) at the zone center [80], which is required for estimating $e_{T}^{*}$. In Table 4, we have reported our calculated values of phonon frequencies at critical points (Γ, X, L, K, and W) at P (=8 GPa), as no experimental and/or theoretical data are available for comparison. Obviously, the $\omega_{j}(\vec{q})$ results reported by Chibueze [91] and Zafar et al. [92] at P = 0 GPa, using ab initio methods, are either ambiguous and/or questionable.

#### 3.2.2. Analyses of the Second-Order P-Dependent Raman Spectra

**Figure 4:**
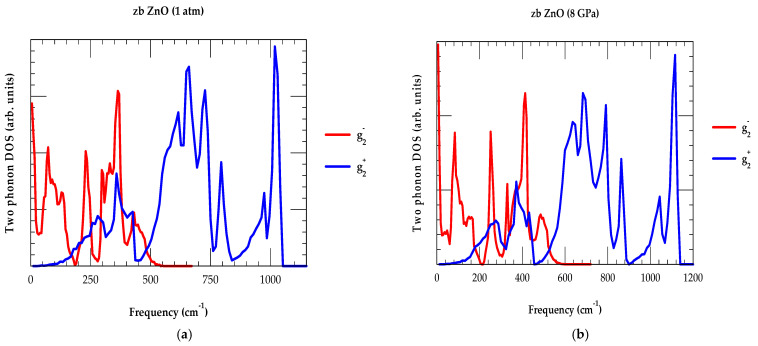
(**a**) At ambient pressure P = 0 GPa, we have reported our RIM calculations for the two-phonon DOS of the zb ZnO material. The $g_{2}^{+}\left(\omega \right)\;$ (sum) of DOS is indicated by using the blue color line and, the $g_{2}^{-}\left(\omega \right)$ (difference) of DOS is shown by red color line. These results are used for analyzing the experimental two-phonon Raman and infrared spectroscopy data on the ZnO material (see text and Table 5). (**b**) Similar results of RIM calculations are shown for the zb ZnO material but at higher pressure P = 8 GPa.

By using the IFCs (cf. Table 3) at ambient and higher P (≡ 8 GPa), we have calculated the two-phonon DOS (i.e., $g_{2}^{+}\left(\omega \right)\;$(sum) and $g_{2}^{-}\left(\omega \right)$ (difference)) for the zb ZnO (see Figure 4a,b), and we interpreted the observed P-dependent Raman features (see Table 5) for the wz material. The phonon spectra of wz ZnO have been studied by Raman scattering [75,76,77,80] and infrared spectroscopy [73]. The IR absorption data, which are mostly connected with the multi-phonon processes and have wave vectors in the vicinity of the BZ edge, are not well-resolved and, therefore, are quite difficult to interpret. On the other hand, the RSS measurements lack polarization data and, therefore, cannot unambiguously designate the crystal symmetry. 

The justification to analyze the second-order Raman scattering spectroscopy data of the wz ZnO (Table 5) by using two-phonon $g_{2}^{+}\left(\omega \right)\;$(sum) and $g_{2}^{-}\left(\omega \right)$ (difference) DOS of zb ZnO is simply related to the fact that the frequencies of major high-critical points of the zb and wz modifications have the same atomic surroundings for the first-nearest neighbors. In the wz crystal, while the sublattice stacking has been designated as ABABAB… along the c-axis, the zb showed a different arrangement of ABCABC… along the <111> direction [79,80,81]. The fact that the wz primitive cell has four atoms along the c-axis allowed us to consider phonon dispersion relations of the wz structure along the Γ-A direction, as backfolded from those of the Γ-L direction in the zb BZ.

### 3.3. Thermodynamic Characteristics

For different materials, the Debye temperature $\Theta_{D}\left(T\right)$ is a valuable physical parameter frequently used for comprehending the excitation of phonons in assessing their interatomic forces. The heat capacity $C_{V}\left(T\right)$ of a solid is a strong measure of the thermal energy stored in all active oscillations of the atoms. In semiconductors, the value of $\Theta_{D}\left(T\right)$ is frequently obtained by exploiting either the speed-of-sound and/or low-temperature heat capacity $C_{V}$ measurements [22,103]. No experimental values of $\Theta_{D}$ and $C_{V}$ are available for the zb ZnO material. However, the importance of these quantities has played a valuable role in its selection as a material in designing several device structures for diverse application needs [1,2,3,4,5,6,7,8,9,10,11,12]. By incorporating $\omega_{j}(\vec{q})$ and $g\left(\omega \right)$ in Equations (11)–(13), we have calculated the specific heat at constant volume $C_{V}\left(T\right)$ and carefully assessed the values of $\Theta_{D}\left(T\right)$ for the zb ZnO.

#### 3.3.1. Debye Temperature of zb ZnO

**Figure 5:**
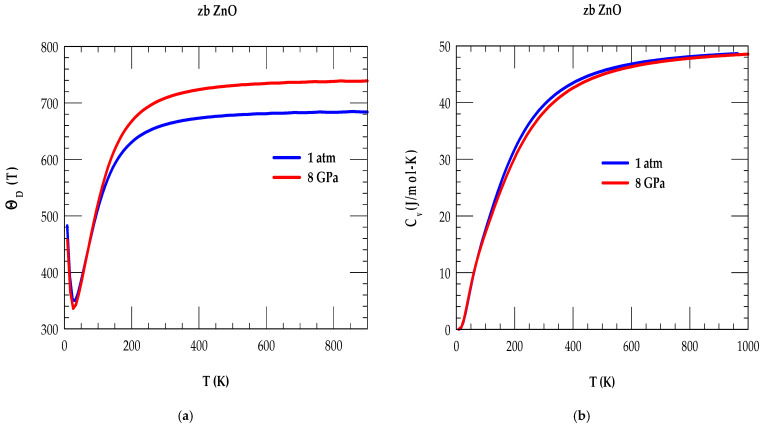
(**a**) Rigid-ion model calculations of the Debye temperature for the zinc-blende ZnO material as a function of T. The full blue- and red-color lines represent our calculated results at 1 atm or P = 0 and P = 8 GPa, respectively. (**b**) The rigid-ion model calculations of specific heat at constant volume for the zb ZnO as a function of T. Solid blue- and red-color lines represent our results at 1 atm or P = 0 and P = 8 GPa.

The simulated results of $\Theta_{D}\left(T\right)$ as a function of T between (≡0 to 900 K) are displayed in Figure 5a at ambient (i.e., P = 0 GPa) and higher pressure (P = 8 GPa) using blue- and red-color lines, respectively. At P = 0, our RIM calculation provided a value of $\Theta_{D}\left(T\to 0\right)$ ~ 487 K, at nearly 0 K, which attained a minimum $\Theta_{D}^{min}\left(T\right)\;\sim \;350\;K$ at ~32 K and reached a higher $\Theta_{D}\left(297\right)$ 661 K at RT, respectively. Although no experimental measurements are known for zb ZnO, the results agree reasonably well with the values reported in the literature [22,103] for different polymorphs. Again, in the absence of P-dependent Debye temperature measurements, our study at P = 8 GPa for zb ZnO has predicted a slight decrease in $\Theta_{D}\left(T\to 0\right)\;$~ 466 K at 0 K, while attaining $\Theta_{D}^{min}\left(T\right)\;\sim \;337\;K$ at 27 K and achieving $\Theta_{D}\left(297\right)$ 707 K, respectively. The simulated trends in zb ZnO material have agreed reasonably well with the values attained in many II-VI compound semiconductors [22,103]. In $\Theta_{D}\left(T\right)$, the dip at low T and rise at higher T can be attributed to the mode-softening and stiffening of the low-frequency acoustic $\omega_{TA(X)};\omega_{TA(L)}$ and high energy $\omega_{LA};\omega_{TO};\omega_{LO}$ phonons, respectively.

#### 3.3.2. Specific Heat of zb ZnO

For the zb ZnO material, we have shown in Figure 5b our simulated T-dependent results of the specific heat at constant volume, i.e., $C_{V}\left(T\right)$ 0 ≤ T ≤ 1000 K for ambient 1 atm or P = 0 (blue color) and P = 8 GPa (red-color lines). Again, with respect to P = 0, we have noticed that for temperature T ≥ 44 K, the values of $\Theta_{D}\left(T\right)$ (cf. Figure 5a) become higher at higher P = 8 GPa, which are changed to the lower value of $C_{V}\left(T\right)$ (cf. Figure 5b). These observations are quite acceptable due to the fact that the impact of increasing pressure is like decreasing the temperature [22,103]. Again, for T well-above $\Theta_{D}$ value, one would expect $C_{V}\left(T\right)$ to attain values closer to the Dulong–Petit limit (~50 (J/mol-K)) [22,103]. Again, at higher T, these results in the zb ZnO are justified, as one would anticipate all of the vibrational modes of oscillations to become excited and participate in providing the contributions to $C_{V}\left(T\right)$.

In Table 6, we have summarized our simulated RIM results by comparing them to the existing theoretical and/or experimental data from the literature [22,103] for the Debye temperature of $\Theta_{D}\left(0\right)$, $\Theta_{D}^{min}\left(T\right)$, and $\Theta_{D}\left(297\right)$ in (K); $C_{V}$(297) in (J/mol-K); and α(T) [104,105] in (10^−6^ K^−1^). One must note that the experimental data for α(T) were only reported for wz ZnO material in the late 60s or early 70s. Moreover, our predicted value of the Born’s effective charge $e_{T}^{*}$ at ambient P compared reasonably well to an earlier [80] result. To extract P-dependent $e_{T}^{*}$ from $\Delta \omega_{opt}$(≡$\omega_{LO(\Gamma )}$ − $\omega_{TO(\Gamma )}$), one needs to have $\varepsilon_{\infty }$ at higher P. In the absence of this value at P = 8 GPa, our calculation has retained $\varepsilon_{\infty }$ at ambient P.

#### 3.3.3. Mode Grüneisen Parameters 

**Figure 6:**
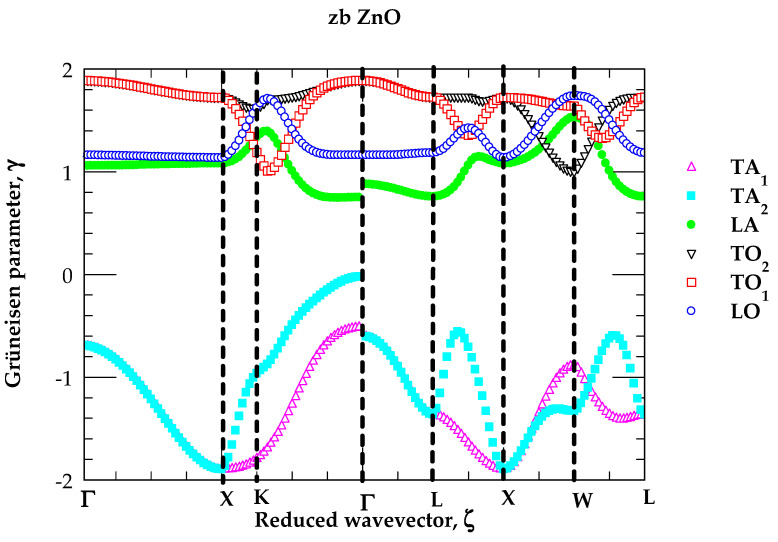
Rigid-ion model calculations of mode Grüneisen dispersions $\gamma_{j}\left(\vec{q}\right)$ of zinc-blende ZnO as a function of the reduced wave vector, ζ.

In Figure 6, for zb ZnO, we report our simulated results of the mode Grüneisen parameters $\gamma_{j}\left(\vec{q}\right)$ along the high symmetry directions in the BZ following the methodology outlined in Section 2.2.3.

Except for zb ZnO material, the P-dependent Raman measurements in many semiconductors [107,108,109,110,111] have confirmed observing the negative (positive) values of $\gamma_{j}\left(\vec{q}\right)$, i.e., the weakening (strengthening) of low (high)-energy phonons [112,113]. In Table 4, we have reported our RIM results of the linear pressure coefficients $a_{j}^{P}$ (≡$\;\partial \omega_{j}(\vec{q})/\partial P$) and $\gamma_{j}\left(\vec{q}\right)$ for zb ZnO. The mode γs at critical points are compared to the limited experimental [79,80] and first-principles results [80]. In many tetrahedrally bonded (e.g., IV-IV, III-V, II-VI, and I-VII) materials [107,108,109,110,111,112,113,114,115,116,117], it has been confirmed experimentally that the mode-softening of $\gamma_{TA(X,L)}$ is caused by compression, which triggers the negative tension in their bonds due to the increased repulsion of the electron-charge overlaps. This negative tension is responsible for significantly modifying the atomic bonding characteristics which drive the phase transitions [107,108,109,110,111,112,113,114,115,116,117]. From Figure 6 and Table 4, one can see the mode Grüneisen parameters of $\omega_{TA}$ phonons exhibiting large negative values in sharp contrast to the positive values of the longitudinal acoustic $\omega_{LA}$ and the optical $\omega_{LO};\omega_{TO}$ modes of zb ZnO. One, therefore, expects that these negative $\gamma_{TA}\left(\vec{q}\right)$ values are responsible for causing NTE at low T, provided the large softening of $\gamma_{TA}$ modes dominate over the stiffening of high frequency $\gamma_{j}\left(\vec{q}\right)$ ($\omega_{LA}$, $\omega_{LO}$, $\omega_{TO}$) modes. 

#### 3.3.4. Thermal Expansion 

**Figure 7:**
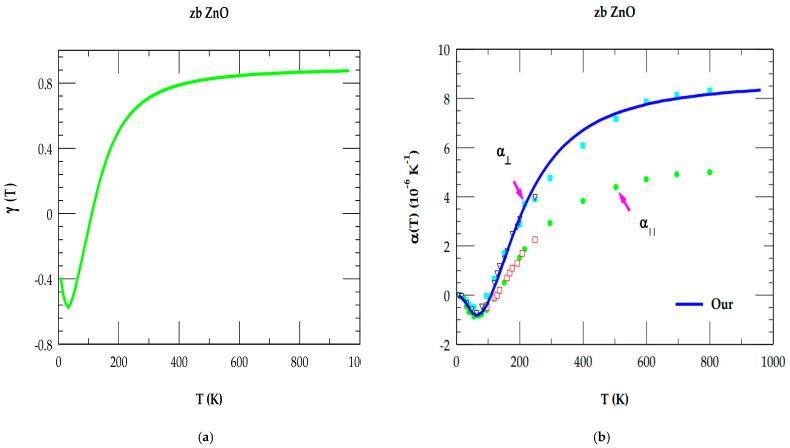
(**a**) Rigid-ion model calculations of the Grüneisen constant γ(T) as a function of T. (**b**) Comparison of rigid-ion model calculations (blue-color line) of linear thermal expansion coefficient α(T) as a function of T for zinc-blende ZnO with the data available for wz material. Ref. [104]: Hollow triangles and hollow squares; Ref. [105]: blue square and green dot.

In Section 3.3.3, we have reported our simulated mode Grüneisen parameters $\gamma_{j}\left(\vec{q}\right)$ along the high symmetry directions. Using Equations (8)–(11), the results of the T-dependent average Grüneisen constant γ(T) and the linear thermal expansion coefficient α(T) are displayed, respectively, in Figure 7a,b for 0 ≤ T ≤ 1000 K. Obviously, these results in the zb ZnO have established an important fact, which is that the negative γ(T) at low T (see Figure 7a) provided a direct correlation to the NTE α(T) (see Figure 7b). 

Several experimental studies on the elemental IV and III-V, II-VI, and I-VII compound semiconductors [107,108,109,110,111,112,113,114,115,116,117] with NTE at low T have exhibited negative values of $\gamma_{TA(X)}$ in the vicinity of the X critical point in the BZ. For zb ZnO, our simulated results of $\gamma_{j}\left(\vec{q}\right)$ (see Table 4) are comparable to many III-V and II-VI semiconductors [102]. There are no experimental results available for comparison in zb ZnO. However, in the QHA only one first-principles DFT study exists of $\alpha_{V}(T)$ (0 ≤ T ≤ 160) [98] for the wz- and zb-structured ZnO material. In Figure 7b, we show our simulated results of the linear thermal expansion α(T) and compare them to the experimental results known for the wz material [104,105]. The agreement of our results with experimental [104,105] and theoretical results [98] is quite encouraging.

## 4. Concluding Remarks

In conclusion, we have performed realistic RIM [99] calculations for the technologically important zb ZnO material, whose transport properties are typically dominated by its atomic vibrations and thermodynamic characteristics. At the ambient 1 atm or pressure of P = 0 GPa, our methodical study has provided accurate phonon dispersions $\omega_{j}(\vec{q})$. These results are in very good agreement with the Raman scattering spectroscopy [80] and first-principles calculations [89]. At higher pressure (P = 8 GPa), the predictions are made for the $\omega_{j}(\vec{q})$, as well as the T-dependent $\Theta_{D}\left(T\right)$, and $C_{v}\left(T\right).$ These results have helped us in calculating $\gamma \left(T\right)$ and $\alpha \left(T\right)$ in the QHA. We strongly believe that the large negative values of $\gamma_{TA}\left(\vec{q}\;\neq \;0\right)$ for the zb ZnO are responsible for the NTE at low temperature (T ≤ 0.2 $\Theta_{D}\left(T\to 0\right))$ [115]. In the LDH based thermal management systems, thermal expansion coefficient $\alpha \left(T\right)$ of different solids plays an important role [1,2,3,4,5,6,7,8,9,10,11,12]. By pairing materials with positive and negative coefficients of thermal expansion, it is possible to create a balanced structure to achieve the necessary conditions for managing heat dissipation to minimize the stress on electronic devices. In a (BeO)_m_/(ZnO)_n_ superlattice structure, by carefully adjusting the number of layers (m, n) with positive and negative thermal expansion coefficients, it is likely that one could achieve a near-zero $\alpha \left(T\right)$. Such a nanostructure might experience a minimal dimensional change with the temperature fluctuations, making it ideal for the electronic devices where a precise dimensional stability is vital. We hope that our projected theoretical results with important trends in the structural, elastic, phonon, thermodynamic, and mechanical characteristics of the novel zb ZnO material will encourage experimentalists to perform similar measurements to check our theoretical conjectures.

## Figures and Tables

**Table 1:** The transition pressures and relative volume changes for wurtzite ZnO to rock-salt (B_4_ → B_1_), zinc-blende to rock-salt (B_3_ → B_1_), and wurtzite to zinc-blende (B_4_ → B_3_) structures, Ref. [59].

Material	$P_{t}$^(a)^ $\mathrm{and}\;∆V_{t}$$/V_{0}$	B_4_ → B_1_^(a)^	Others (See Ref. [59])	B_3_ → B_1_^(a)^	Others (See Ref. [59])	B_4_ → B_3_^(a)^	Others (See Ref. [59])
ZnO	$P_{t}$ (GPa)	12.70	6.6–10.1	11.81	2.0–14.50	4.55	3.22–26 ^(b)^
	$∆V_{t}$/$V_{0}$ (%)	24.50, 20.20	15.5–18	24.51, 20.21	17.7–19.42	24.64, 26.40	

^(a)^ Ref. [59] (and references cited therein); ^(b)^ Ref. [60].

**Table 2:** Critical point phonon frequencies (in cm^−1^); lattice constants a_0_ (in Å); elastic constants $c_{ij}$ in (10^11^ dyn/cm^2^); and bulk modulus $B_{0}$ (in GPa) for zb ZnO material. These quantities, along with the pressure derivatives of bulk modulus $B_{0}^{\prime }$ and elastic constants $c_{ij}^{\prime }$, are used to evaluate the optimized set of rigid-ion model (RIM) parameters (see Table 3).

zb ZnO		
	Our	Others ^(a), (b)^
$\omega_{LO(\Gamma )}$	558	525, 517
$\omega_{TO(\Gamma )}$	403	403, 367
$\omega_{LO(X)}$	551	555, 495
$\omega_{TO(X)}$	487	444, 442
$\omega_{LA(X)}$	269	268, 270
$\omega_{TA(X)}$	128	80, 121
$\omega_{LO(L)}$	561	
$\omega_{TO(L)}$	443	
$\omega_{LA(L)}$	264	
$\omega_{TA(L)}$	93	
a_o_	4.504	4.520–4.666
$c_{11}$	19.19	15.1–19.3
$c_{12}$	14.79	11.06–15.8
$c_{44}$	7.34	7.4–12.8
$B_{0}$	162.6	
$B_{0}^{\prime }$	4.0	3.3–4.3
P_t_	11.8	2.0–11.87
$c_{11}^{\prime }$	4.63	
$c_{12}^{\prime }$	3.01	
$c_{44}^{\prime }$	0.33	

^(a)^ Refs. [80,89]; ^(b)^ Refs. [59,60].

**Table 3:** Optimized set of rigid-ion model parameters (10^5^ dyn/cm) for zb ZnO at P = 0 and P = 8 GPa in the notation of Ref. [99]. The term Z_eff_ is the effective charge.

RIM ^(a)^	zb ZnO	
Parameters	P = 0 GPa	P = 8 GPa
A	−0.40207	−0.48198
B	−0.395	−0.486
C_1_	−0.0540	−0.0590
C_2_	−0.0490	−0.0520
D_1_	−0.0088	−0.01193
D_2_	−0.0900	−0.0880
E_1_	−0.0300	−0.05000
E_2_	0.0600	0.09000
F_1_	−0.0360	−0.0400
F_2_	0.12300	0.15300
Z_eff_	0.9435	0.8411

^(a)^ Ref. [99]

**Table 4:** Comparison of the critical point phonon frequencies (cm^−1^) obtained at P = 0, for zb ZnO by rigid-ion model (RIM) with the ab initio calculations and Raman scattering spectroscopy measurements from the literature. The RIM phonons at P = 8 GPa and linear pressure coefficients $a_{j}^{P}$ (cm^−1^/GPa) and Grüneisen parameters $\gamma_{j}\left(\vec{q}\right)$ are also reported and compared with existing data [80].

Modes zb ZnO	Our RIM ^(a)^ P = 0	Ab initio Calc. ^(b,c)^ P = 0	Raman Data ^(d)^ P = 0	Others ^(e)^ P = 0	Our RIM ^(a)^ P = 8	$a_{j}^{P}=\frac{\partial \omega_{j}}{\partial P}$ RIM ^(a)^	$\gamma_{j}$ RIM ^(a)^	$\gamma_{j}$ Others ^(d)^
$\omega_{LO(\Gamma )}$	558	525, 517	558	379	598	5	1.16	1.31
$\omega_{TO(\Gamma )}$	403	403, 367	403	379	450	5.9	1.89	2.03
$\omega_{LO(X)}$	551	555, 495	551	523	590	4.9	1.14	1.37
$\omega_{TO(X)}$	481	444, 442	487	466, 418	532	6.4	1.72	1.85
$\omega_{LA(X)}$	269	268, 270	269	223	287	2.3	1.08	1.18
$\omega_{TA(X)}$	127	80, 121	128	144, 93	112	−1.9	−1.89	−1.24
$\omega_{LO(L)}$	558		561	536	598	5	1.18	1.42
$\omega_{TO(L)}$	455		443	417	504	6.1	1.73	1.99
$\omega_{LA(L)}$	268		264	257	281	1.6	0.76	0.88
$\omega_{TA(L)}$	87		93	90	80	−1.0	−1.36	−1.71
$\omega_{LO(K)}$	541				595	6.8	1.62	
$\omega_{TO1(K)}$	481				527	5.8	1.19	
$\omega_{TO2(K)}$	476				524	6	1.62	
$\omega_{LA(K)}$	249				270	2.6	1.33	
$\omega_{TA1(K)}$	176				165	−1.38	−0.96	
$\omega_{TA2(K)}$	115				102	−1.6	−1.79	
$\omega_{LO(W)}$	539				597	7.3	1.74	
$\omega_{TO1(W)}$	487				536	6.1	1.64	
$\omega_{TO2(W)}$	485				515	3.8	1	
$\omega_{LA(W)}$	242				265	2.9	1.53	
$\omega_{TA1(W)}$	174				159	−1.9	−1.33	
$\omega_{TA2(W)}$	142				134	−1.0	−0.88	

^(a)^ Ours; ^(b)^ Ref. [92]; ^(c)^ Ref. [89]; ^(d)^ Ref. [80]; ^(e)^ Ref. [91].

**Table 5:** Comparison of structures in the two-phonon DOS sum and difference modes with the second-order Raman data [75,76,77,80].

Mode	Our Calculations	Experiments
2LO (Γ)	1196	1200 ^(a), (b)^
LO + TO(X)	1122	1149, ^(a)^ 1160 ^(b)^
LO + TO(L)	1102	1084, ^(a)^ 1080 ^(b)^
2 TO(L)	1008	986, ^(a)^ 990 ^(b)^
TO + LA(X)	785	800–820, ^(a)^ 810 ^(b)^
LO + TA	678	675, ^(a)^ 680 ^(b)^
TO + TA(L)	584	Overlaps with first order
2LA(K)	540	540, ^(a)^ 530 ^(b)^
LO-LA(K)	325	334, ^(a)^ 330, ^(b)^ 331 ^(c)^
LO-LA(X)	303	302 ^(d)^
2TA(X)	224	208, ^(a)^ 205, ^(b)^ 213 ^(c)^

^(a)^ Ref. [75]; ^(b)^ Ref. [76]; ^(c)^ Ref. [77]; ^(d)^ Ref. [80].

**Table 6:** The RIM calculations of Debye temperature $\Theta_{D}\left(0\right)$, $\Theta_{D}^{min}\left(T\right)$, $\Theta_{D}\left(297\right)$ in (K); $C_{V}$(297) in (J/mol-K); α(T) in (10^−6^K^−1^); and $e_{T}^{*}$ for zb ZnO are compared to existing experimental and/or theoretical data from the literature.

zb ZnO					
Quantity	RIM, P = 0 GPa ^(a)^	Others ^(b)^	Others ^(c)^		RIM, P = 8 GPa ^(a)^
			$\alpha_{┴}$	$\alpha_{\left|\right|}$	
$\Theta_{D}\left(0\right)$	487				466
$\Theta_{D}^{min}\left(T\right)$	350 @ 32 K				337 @ 27 K
$\Theta_{D}\left(297\right)$	661				707
$C_{V}(297)$	39.43	40.0, 41.84			38.4
α(100)	−0.41		0.04	−0.62	
α(200)	3.16		2.88	1.51	
α(300)	4.37		4.75	2.92	
α(800)	8.17		8.3	4.98	
$e_{T}^{*}$	2.16	2.13			1.99

^(a)^ Ours; ^(b)^ Refs. [22,80,103]; ^(c)^ Refs. [104,105].

## Data Availability

The data that support the findings of this study will be available from the corresponding author upon reasonable request.
